# Potential benefits of kefir and its compounds on Alzheimer's disease: A systematic review

**DOI:** 10.1016/j.bbii.2025.100115

**Published:** 2025-04

**Authors:** Yuri Castelo Branco Tanure, Ana Clara Morais Mafra, Bruna Luiza Motta Guimarães, Rafael Coelho Magalhães, Catherine Fagundez, Israel Júnior Borges do Nascimento, Julio Cesar Moreira Brito

**Affiliations:** a Fundação Ezequiel Dias (FUNED), Belo Horizonte, MG, Brazil; b Universidade Federal de Minas Gerais (UFMG), Belo Horizonte, MG, Brazil; c Universidad de la República, Montevideo, Uruguay; d Division of Country Health Policies and Systems (CPS), World Health Organization, Regional Office for Europe, Copenhagen 2100, Denmark

**Keywords:** Kefir, Fermented milk, Neurodegenerative diseases and Alzheimer

## Abstract

Alzheimer's disease, characterized by the progressive loss of cognitive functions of the brain, is still an incurable pathology. Current treatments primarily aim to alleviate symptoms, acting mainly on behavioral changes, having a modest impact in the disease course. Recently, potential role of probiotics in managing Alzheimer's has been explored. Kefir, a fermented food teeming with live microorganisms, is thought to influence the gut microbiota, potentially reducing inflammation and the accumulation of toxic proteins in the brain. Additionally, kefir contains bioactive compounds, such as B vitamins, choline, and folic acid, which are essential for neuronal health and cognitive function. Thus, kefir could emerge as a promising complementary treatment for Alzheimer's disease. This systematic review, conducted in January 2024, examined the effects of kefir in both in vivo animal models and human patients with neurodegenerative conditions. The review was based on studies retrieved from BVS, Embase, PubMed/MEDLINE, Scopus, and Web of Science databases. Seven studies were included, involving invertebrates, murine models, and human participants. In animal models, the primary outcomes were antioxidant effects, reduced beta-amyloid deposition, and attenuation of vascular damage and neurodegeneration. In human studies, kefir supplementation resulted in decreased levels of inflammatory cytokines, reactive oxygen species (ROS), and oxidative proteins, and was associated with improvements in memory. Given its potential benefits, kefir could serve as a valuable adjunct to conventional treatments for Alzheimer's disease, warranting further investigation in clinical settings.

## Introduction

Alzheimer's disease (AD) is a progressive neurodegenerative condition which is the main cause of dementia in the world, posing a significant challenge in healthcare nowadays due to the aging of the world population. It is characterized primarily by a decline in cognitive function and memory loss. These symptoms typically manifest in advanced ages and have a significant progression over time (Schilling et al., 2022).

This disease is often associated with comorbidities such as hypertension, diabetes, obesity and dyslipidemia, and it’s mechanism, although widely studied and described, may be influenced by many factors not yet fully elucidated. There is no cure to AD and the pharmacological treatment currently aims at controlling cognitive and behavioral symptoms as the disease is already established. New treatments aim to prevent the formation of the senile plaques, which are responsible for the disease’s course in the most consolidated physiopathological model. In addition, lifestyle changes and dietary approaches are seen as useful strategies in the management of risk factors, being an important form of prevention of dementia *(*Livingston et al., 2020).

As a complementary proposition to medical and dietary interventions, probiotics have been used willing to improve cognitive symptoms in AD patients by acting on pathways that may contribute to AD physiopathology, such as the enhancement of oxidative stress and neuroinflammation. Many fermented products have been used with this goal, once fermentation process enriches the nutritional contents in the food and its biological properties, including anti-oxidative, anti-inflammatory, anti-apoptotic, and antimicrobial, which can provide health benefits (Kumar et al., 2022). In this field, Kefir stands out among other fermented probiotics due to its unique microbial composition, including both bacteria and yeast in a symbiotic relationship. Additionally, kefir is adaptable to various substrates, allowing for the production of both dairy and non-dairy variants, which makes it accessible for people with different dietary needs (Azizi et al., 2021, Vieira et al., 2021). Recent researches have shown a strong correlation between AD and intestinal dysbiosis, which lead to new thoughts regarding the pathophysiology of the disease as well as it’s treatment (Liu et al., 2020).

Kefir is an acidic fermented probiotic drink originated in the Caucasus, Eastern Europe and the Balkans, being formed mostly by a matrix of exopolysaccharides in which symbiotic lactic acid bacteria, acetic acid bacteria and yeast are found (Garofalo et al., 2020). It contains a high quantity of proteins and amino acids, unsaturated fatty acids, carbohydrates such as polysaccharides, vitamins A, the B complex, D, E and K, minerals such as calcium, phosphorus, potassium and zinc and a low concentration of lactose (Arslan, 2015).

Recently, kefir grains have garnered attention for their potential therapeutic benefits in AD due to their anti-inflammatory and antioxidant effects, as well as their probiotic properties (Pereira et al., 2021). These properties could potentially reduce neuroinflammation and oxidative stress, both of which are implicated in the progression of AD (Liu et al., 2020).

This review aims to evaluate existing studies that investigate the effects of kefir on AD. By synthesizing current research findings, our objective is to provide a comprehensive analysis of the potential benefits of kefir in reducing cognitive symptoms and neurodegeneration associated with AD.

## Methods

This systematic review and meta-analysis were conducted in accordance with the recommendations of the Cochrane Handbook. The Preferred Reporting Items for Systematic Reviews and Meta-Analyzes (PRISMA) were followed in all phases of searching, selecting, extracting and analyzing the relevant data (Higgins et al., 2023, Page et al., 2021).

Information on the potential therapeutic benefits of kefir in neurodegenerative diseases, particularly AD, was collected through computerized searches in five databases: PubMed/MEDLINE, Scopus, Embase, Virtual Health Library (VHL) and Web of Science.

The analysis included articles describing the pharmacological activity of kefir in neurodegenerative diseases. The preliminary inclusion criteria were articles published in English, Portuguese or Spanish. The searches were conducted on January 8, 2024. Medical Subject Heading (MeSH) was used to define the prescribers included in the database search. The search was conducted as follows: the keywords "neurodegenerative" OR "neurodegenerative diseases" were paired with the term "kefir".

We used the PICOS strategy to find relevant studies among the articles found in the electronic search: "population", which included laboratory animal and human studies with nerodegenerative diseases; "intervention", which included exposure to kefir; "control", which included a comparison between intervention and placebo; "outcomes”, which included clinical changes; and "study design", which included in vivo studies (Amir-Behghadami and Janati, 2020). First, two independent researchers (B.L.M.G. and A.C.M.M.) assessed the titles, abstracts and keywords of the articles based on the PICOS eligibility criteria (Eriksen and Frandsen, 2018). The following exclusion criteria were used for the selection of articles: (i) articles without full text, (ii) notices, (iii) editorials, (iv) letters, (v) notes, (vi) review articles. Articles in languages other than Portuguese, English and Spanish as well as dissertations, abstracts, books, notes and reports were also excluded. The same researchers evaluated the papers and selected only those that met the criteria of the study. Relevant data were extracted for review and analysis.

## Results

### Database search and study selection

**Fig. 1:**
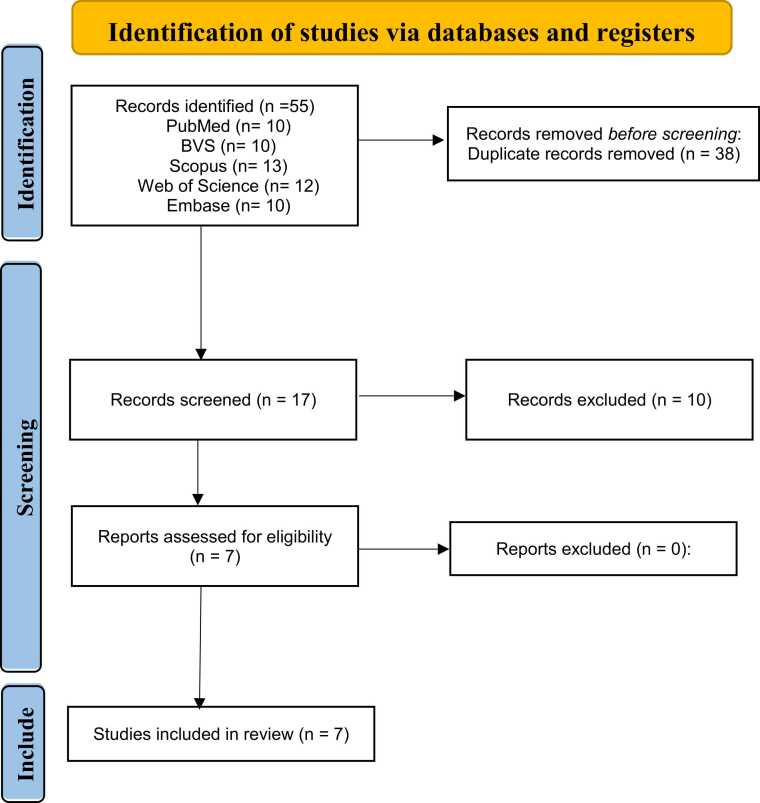
Flowchart of the kefir and Alzheimer's disease articles selection for systematic review and meta-analysis according to PRISMA criteria.

A search of the databases retrieved 55 articles (10 from PubMed/MEDLINE, 13 from Scopus, 10 from BVS, 12 from Web of Science, and 10 from Embase), as shown in Fig. 1. After duplicates were removed, 17 studies were screened for titles, abstracts, and keywords that met the inclusion criteria, and 10 studies were excluded, of which 7 were reviews and 3 studies didn’t meet the design criteria. The kappa agreement index between the researchers who made the selection was 0.769, showing a good strength of agreement (Eriksen and Frandsen, 2018). After reading the entire content of the remaining 7 studies (Anwar et al., 2019, Batista et al., 2021, El Sayed et al., 2021a, El Sayed et al., 2021b, Malta et al., 2022, Ton et al., 2020, Wang et al., 2022), the information presented in Table 1, Table 2, Table 3 was extracted.

**Table 1:** Main characteristics of in vivo studies with included rodents.

**Author**	**Method**	**Analyzed Material**	**Outcomes**
Wang et al. (2022)	✓ 40 ♂ (5 groups of 8 W*R*); ✓ AD rat model (12 weeks continuos i.p. 90 mg/kg/day D-galactose + o.a 40 mg/kg/day AlCl_3_).	✓ Cognitive function by MWM and OFT; ✓ 16S rRNA gene sequencing; ✓ Aβ; ✓ αβ42 in the hippocampus and cortex; ✓ LPS in the feces, brain, and blood; ✓ Blood ammonia; ✓ Metabolomics analysis.	✓ ↓Cognitive deficits, anxiety-like behaviors and neuronal degeneration; ✓ ↓ Aβ accumulation in the brain; and ↓ activation of microglia and neuroinflammation through TLR4/MYD88/NLRP3 signaling pathway; ✓ ↓ intestinal mucosal impairments; ✓ MA2 reshaped the gut microbiota structure, composition, and Remarkably modulated the glycometabolism; ✓ Inhibitory effects on the Aβ42 aggregation and amyloid-induced cytotoxicity.
Anwar et al. (2019)	✓ 60 ♀ (6 groups of 10 AR); ✓ Model (i.p. of 0.56 mg/kg LPS in 1 mL of sterile PBS (pH 7) to induce neuro-inflammatory AD.	✓ Brain tissue cholesterol level; ✓ IL−10 and TNF-α level in brain tissue; ✓ Plasma lipid profile; ✓ Blood GSH and MDA; ✓ TMT; ✓ BM derived mesenchymal stem cells from rats; ✓ Brain total RNA.	✓ ↑ protection against LPS induced changes and ↓ the time spent by rats at TMT; ✓ ↑ memory recovery; ✓ ↓ Bax; ✓ ↑ BDNF, Bcl−2, seladine−1; ✓ ↓ TNF-α ↑ in IL−10 level; ✓ ↓ in tissue cholesterol level; ✓ ↓ cholesterol, LDL and triacylglycerol level; ✓ ↓ oxidative stress; ✓ ↓ dense plaques multiple and less prominent apoptotic nuclei.
El Sayed et al., 2021b	✓ 72 ♂ (6 groups of 12 AM); ✓ AD mice model (ICV STZ 3 mg/kg dissolved in 0.9 % saline).	✓ NORT and MWM; ✓ ELISA: Ach, Aβ1–42, p-NF-κB, TNF-α, IL6, NLRP3, and IDE in the hippocampus; ✓ Colorimetric: hippocampal MDA and GSH; ✓ Western Blot: p-PI3K, p-Akt, p-GSK−3β, p-mTOR, and p-tau protein; ✓ Expression in the hippocampus; ✓ RT-PCR// analysis of insulin Receptor, RAGE, and TLR4; ✓ Gene expression in the hippocampus; ✓ Histopathological examination.	✓ Improved Ach level hippocampal; ✓ ↓ Aβ1–42 content; ✓ Preserved neurons in the hippocampus and the cerebral córtex; ✓ ↑ Insulin signaling and regulation of insulin receptor expression and augmentation of PI3K/Akt signaling; ✓ Modulation of GSK−3β and mTOR activity; ✓ ↓ Accumulation of p-tau; ✓ ↓ Oxidative stress,inflammation and neurodegeneration; ✓ Protected the neurons in the hippocampus and the cerebral cortex from inflammation-induced death; ✓ Restoration of the gut microbiome; ✓ Significant downregulation in the expression of TLR4.
El Sayed et al., 2021a	✓ 72 ♂ (6 groups of 12 AM); ✓ AD mice model (ICV injection of STZ (3 mg/kg) dissolved in 0.9 % saline).	✓ NOR, MWMT and GSH; ✓ Analysis of phosphorylated Tau, GSK−3β, and mTOR hippocampus protein expression; ✓ Levels of Ach, amyloid β1–42, MDA, Nrf−2, HO−1, NF-κB, NLRP3, IL−1β, TNF-α, Caspase−3, and IDE in the hippocampus; ✓ Reaction Analysis of ERK1/2 and p38-MAPK gene expression in the hippocampus; ✓ Determination of protein content; ✓ Histopathological examination.	✓ ↓ Cognitive dysfunction and tau hyperphosphorylation; ✓ ↑ Memory, learning and hippocampal level of IDE; ✓ ↓ Aβ level in the hippocampus; ✓ ↓ AD symptoms comparable to those of simvastatin; ✓ ↑ Ach concentration and ↓activity of caspase−3; ✓ ↓ MDA content; ✓ ↓ neutrophil infiltration, nitrotyrosine formation, NF-κB activation, and inducible nitric oxide synthase expression.

♀ – Female; ♂ – Male; **Aβ** – Amyloid β; **AD** – Alzheimer disease; **AR** – albino rat; **BDNF** – Brain-derived neurotrophic fator; **BM** – bone marrow; **ICV** – intracerebroventricularly; **IDE** – Insulin-Degrading Enzyme; **i.p.** – intraperitoneal injection; **LDL** – low density liprotein; **LPS** – lipopolysaccharide; **MA2** – *Lactobacillus plantarum*; **MDA** – malondialdehyde; **MWM** – Morris watermaze; **NORT** – Novel Object Recognition Test; **o.a**. – oral administration; **RAGE** – Receptor for the Advanced Glycation and Products; **STZ** – Streptozotocin; **TMT** – T Maze Test; **W*R*** – Wistar rats.

**Table 2:** Main characteristics of studies using *Drosophila melanogaster* treated with Kefir filtered grains.

**Author**	**Method**	**Analyzed Material**	**Outcomes**
Malta et al. (2022)	✓ AD fly model: Elav-Gal4 ♀ UAS-BACE, UAS-APP ♂ crossed; ✓ Kefir and UFKF (<10 kDa, >10 kDa); ✓ 0–3 days post egg eclosion.	✓ Acetylcholinesterase inhibition; ✓ RING; ✓ Total antioxidant activity; ✓ Amyloid quantification; ✓ Histopathological analysis.	✓ UFKF < 10 kDa (0.25 mg/mL): ↑↑↑ antioxidant activity, ↑ climbing ability, ↓ Aβ levels; inhibited acetylcholinesterase activity; ✓ UFKF > 10 kDa (0.25 mg/mL), ↑↑ antioxidant activity, ↑ climbing ability, ↓ Aβ levels and acetylcholinesterase activity; ✓ Kefir: ↑ Antioxidant activity, ↑ climbing ability and ↓ Aβ levels.
Batista et al. (2021)	✓ AD-like flies: Elav-Gal4 ♀ UAS-BACE,UAS-APP ♂ crossed; ✓ Kefir or its fractions: Hx, DCM, AcOEt or NBT; ✓ Treated 15 D.P.E.	✓ 16S rRNA gene sequencing; ✓ Beta Amyloid; ✓ Flies’ climbing ability; ✓ Histopathological analysis.	✓ Kefir ↑ the survival rate of AD-like flies; ✓ ↑ Climbing ability; ✓ ↓ Vacuolar lesions; ✓ DCM 0.25 mg/mL: ↓ neurodegenerative index; ✓ Hx; DCM ↑ fly climbing; ✓ AcOEt; nBT ↑ AD-like flies' survival; ✓ ↑ antioxidant, anti-inflammatory, and anti-BACE; ✓ n-butOH: ↑ ↑ ↑ AD- like fies’ climbing ability, ↓ vacuolar lesions.

♂ – Male; ♀ – Female; AcOEt - Ethyl acetate; AD – Alzheimer disease; Aβ - Amyloid β; DCM – Dichloromethane; D.P.E - Days Post Eclosion; Hx – Hexane; kDa – kilodalton; nBT - n-butanol; RING- Rapid iterative negative geotaxis assay; UFKF – Ultrafiltration kefir fraction.

**Table 3:** Main characteristics of clinical study included.

**Author**	**Method**	**Analyzed Material**	**Outcomes**
.Ton et al., 2020	✓ 13 patients; ✓ Kefir supplementation at the daily dose of 2 mL per kilogram of body weight; ✓ Global cognitive functions – MMSE; ✓ Memory - immediate memory test and delayed memory test; ✓ Visual-spatial and abstraction abilities (Cookie Theft Picture Test); ✓ Executive and language functions (Boston Naming Test); ✓ Verbal fluency test (Trail Making Test); ✓ Attentive function (Trail Making Test); ✓ Visuoconstructive abilities (Clock-drawing test).	✓ Cytokines; ✓ ROS; ✓ MMP; ✓ p53; ✓ Cleaved PARP Expression ✓ Cell Cycle Analysis ✓ Cell Viability and Apoptosis	✓ ↑ Performance in the MMSE in 28 %; ✓ ↑ Memory analysis - immediate memory test and late memory test (66 % and 62 %); ✓ ↑ Visual-spatial and abstraction abilities; ✓ ↑ Boston Test and verbal fluency test (30 %, and 25 %); ✓ ↑ constructive abilities; ✓ ↓ TNF-α, IL−8, and ↓ IL12p70; ✓ ↓ Serum levels of O -, H2O2, and ONOO /OH with a ↑ in NO levels ✓ ↓ Protein oxidation; ✓ ↓ ROS production and recovery of mitochondrial membrane potential (MMP); ✓ ↑ p53 expression; ✓ ↑ G0/G1 phase; ✓ ↓ % of cells in S/G2/M phases; ✓ ↓ Cells with sub-G DNA content after kefir treatment; ✓ ↓ DNA fragmentation; ✓ ↓ Cleaved PARP−1; ✓ ↑ Memory, language, executive functions, visual-spatial function, conceptualization, and abstraction abilities; ✓ ↓ Cognitive deficits and anxiety-like behaviors.

**MMSE** – Mini-Mental State Examination; **ROS** – Reactive oxygen species; **MMP** – Mitochondrial Membrane Potential; **PARP** – Poly (ADP-ribose) polymerase.

Four of the selected studies (4/7; 57.14 %) were performed on rodents, totaling 244 animals. These rodents were divided into Wistar rats (40/244; 16.39 %) (Wang et al., 2022), albino rats (60/244; 24.59 %) (Anwar et al., 2019), and albino mice (144/244; 59.01 %) (El Sayed et al., 2021a, El Sayed et al., 2021b), as shown in Table 1. The studies analyzed cognitive function change, neuroinflammation, and brain tissue characteristics. In the first study (Wang et al., 2022), the main outcomes included behavioral assessments and histopathological examination of brain tissue to evaluate the effects of MA2 on AD-induced damage. The second study (Anwar et al., 2019) assessed neuroinflammatory markers, bone marrow-derived stem cell (MSC) integration, and the effects of kefir and MSCs combined, alongside cognitive performance tests. The third study (El Sayed et al., 2021a) focused on the impact of pioglitazone and a kefir product on AD progression, specifically looking at memory, inflammation, and neuronal protection, with different dosage groups for Probiotics Fermentation Technology (PFT). The fourth study (El Sayed et al., 2021b) primarily investigated the effects of simvastatin and a kefir product on cognitive function and brain tissue integrity, with a particular emphasis on oxidative stress, neuroinflammation, and neuronal apoptosis.

Two studies (2/7; 28.57 %) were conducted on *Drosophila melanogaster* flies, as shown in Table 2, and in both studies, amyloid quantification was performed to assess the expression of human amyloid precursor protein (APP) and β-secretase (BACE) in the flies. The first study (Malta et al., 2022) used Thioflavin T staining to directly visualize amyloid deposits, while the second study (Batista et al., 2021) focused on evaluating the effects of kefir on neurodegeneration. Both studies employed the Rapid Iterative Negative Geotaxis (RING) assay to assess motor function decline, a critical indicator of neurodegeneration, through the climbing ability of the flies. Additionally, histopathological analysis was carried out to evaluate the severity of neurodegeneration, using a vacuolar lesion scale to quantify damage in the central brain. Overall, both studies integrated biochemical, behavioral, and histological techniques to assess the progression of AD-like pathology in *Drosophila melanogaster*, with a focus on validating the neurodegenerative phenotype.

One human study (1/7; 14.28 %) was an uncontrolled clinical trial conducted on 13 patients of both sexes, without age restriction, that evaluated patients with Alzheimer's Disease (AD) through convenience sampling. Inclusion criteria involved patients without neurological or psychiatric comorbidities associated with cognitive impairment, without clinical depression and without multiple clinical comorbidities. The intervention consisted of kefir-fermented milk supplementation for 90 days, with cognitive functions, inflammatory biomarkers, oxidative stress, and cellular parameters assessed before and after the intervention (Ton et al., 2020), as summarized in Table 3.

### Cognitive effects

In rodent studies there was a significant decrease in anxious behavior and improvement in T-maze behavior test in one study (Wang et al., 2022), indicating positive cognitive effects. It was also seen an improval in learning ability and memory enhancements in other 2 studies (Anwar et al., 2019, El Sayed et al., 2021b).

In the *Drosophila melanogaster* studies it was noticed an improvement in the survival rate of the subjects (Batista et al., 2021). The treatment with the n-butanol fraction of kefir also resulted in an improvement in parameters such as climbing ability, which is used to assess neurodegeneration in this AD model (Batista et al., 2021, Malta et al., 2022).

In the clinical study, key cognitive assessments revealed enhanced global cognitive status (MMSE), memory performance (both immediate and delayed), visual-spatial and abstraction abilities, as well as executive and language functions. Specifically, the Mini-Mental State Examination (MMSE) showed an improvement of 28 %, and memory tests demonstrated approximately 66 % and 62 % improvements in immediate and late memory, respectively. Additionally, improvements were observed in tests assessing constructive abilities and attention (Ton et al., 2020).

### Antioxidant effects

Antioxidant activity was a significant finding in five studies (71.42 %) (Anwar et al., 2019, Batista et al., 2021, El Sayed et al., 2021a, El Sayed et al., 2021b, Malta et al., 2022, Ton et al., 2020). In rodent studies, kefir treatment reduced oxidative damage by decreasing nitrotyrosine formation and inducible nitric oxide synthase expression (El Sayed et al., 2021b). Similarly, both aqueous and organic kefir fractions exhibited strong antioxidant properties in *Drosophila melanogaster* (Malta et al., 2022).

### Effects on beta-amyloid and Tau proteins

Reduction in beta-amyloid protein accumulation in the brain was highlighted in four studies (57.17 %) (Anwar et al., 2019, El Sayed et al., 2021a, El Sayed et al., 2021b, Malta et al., 2022). Additionally, reductions in hyperphosphorylation of Tau protein were observed in two studies (28.57 %) (El Sayed et al., 2021a, El Sayed et al., 2021b). These findings underscore kefir’s potential in addressing hallmark pathologies of Alzheimer’s disease.

### Neuroinflammatory and neurodegenerative effects

Kefir treatment was associated with reductions in neuroinflammation in three studies (42.85 %) (Batista et al., 2021, El Sayed et al., 2021a, El Sayed et al., 2021b, Wang et al., 2022). Specific findings included decreased NF-κB activation and caspase-3 activity, indicating lower levels of inflammation and apoptosis (El Sayed et al., 2021b). Neurodegeneration was also mitigated in three studies (42.85 %) (Batista et al., 2021, El Sayed et al., 2021a, Wang et al., 2022), with evidence of hippocampal and cortical neurons preservation (El Sayed et al., 2021a). The clinical study demonstrated a reduction in the proportion of microglial cells in the S/G2/M phases, potentially linked to improved cellular function and cognition (Ton et al., 2020).

One of the rodent studies showed improvement in the reduction level of brain tissue BDNF, Bcl2, seladine-1 with decrease in Bax relative expression with variations in the percent of change among the groups in the brain tissue increased protection against LPS-induced changes and a decrease in dense-core amyloid plaques (Anwar et al., 2019).

### Glycometabolism modulation

Three studies (42.85 %) suggested kefir’s potential in modulating glycometabolism (Anwar et al., 2019, El Sayed et al., 2021a, Wang et al., 2022). Improvements included enhanced Insulin degrading enzyme (IDE) levels in the hippocampus and modulation of pathways such as Glycogen synthase kinase (GSK-3β) and mammalian target of rapamycin (mTOR) (El Sayed et al., 2021a, El Sayed et al., 2021b).

### Cytokine and immune modulation

Changes in cytokine profiles were observed in two studies (28.57 %), which demonstrated increased proinflammatory TNF-α and decreased IL-10 and IL-12 levels (Anwar et al., 2019, Ton et al., 2020). Restoration of the intestinal mucosa, noted in two studies (28.57 %) (El Sayed et al., 2021a, Wang et al., 2022), further highlights kefir’s immune-modulatory potential.

## Discussion

AD is the leading cause of dementia worldwide and accounts for about 60 % of irreversible forms of dementia (Schilling et al., 2022). The significance of this disease is increasing as the world's population ages. It is estimated that by 2050 around 150 million people will be affected by AD (Scheltens et al., 2021). Initial symptoms of AD include short-term memory loss, language difficulties and temporal-spatial disorientation. As the disease progresses, patients may develop behavioral changes, severe confusion, disinterest in daily activities and eventually loss of autonomy in daily living activities, highlighting the severe personal and social impact of this disease (Scheltens et al., 2021).

### Pathophysiology and diagnosis

**Fig. 2:**
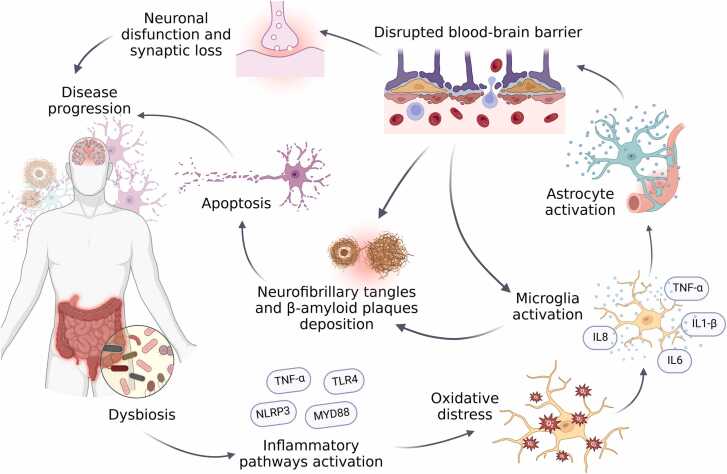
This figure illustrates the role of intestinal dysbiosis in neuroinflammation and the pathophysiology of Alzheimer's disease. Dysbiotic gut microbiota activates inflammatory pathways, leading to increased oxidative stress. This, in turn, triggers microglial activation in the central nervous system. Activated microglia then stimulate astrocytes, resulting in the disruption of the blood-brain barrier (BBB), which further perpetuates microglial activation in a vicious cycle. Both BBB compromise and microglial activation contribute to the formation and deposition of beta-amyloid plaques and neurofibrillary tangles, through hyperphosphorylation of tau protein, which promote neuronal apoptosis, synaptic dysfunction, and disease progression. Created in https://BioRender.com.

In the last decades many hypotheses were developed to explain the neurodegeneration and cognitive impairment in AD progression. The amyloid cascade hypothesis, one of the most promising proposals, points the accumulation of the beta-amyloid protein in plaques as the initial event on the AD pathogenesis, which in addition to the deposition of Tau neurofibrillary tangles, promotes neuronal death and progressive synaptic dysfunction, leading to brain atrophy seen in advanced stages of the disease(Scheltens et al., 2021). However, it was noticed limitations on this model as a single explanation to the disease course, once beta amyloid protein deposition can be found in approximately 30 % of elderly people without cognitive impairment, suggesting that the clearance of the protein may not have satisfactory results as a unique treatment(Rowe et al., 2010). As a result,new researches started to point the influence of many other factors in AD, adapting the theory from a neuron-centric linear cascade to an integrative model, as shown in Fig. 2, which involves many factors as compromised vasculature, disruption of the brain-blood barrier, oxidative stress, microgliosis and dysregulation of proteolysis mechanisms (Liu et al., 2020).

The presence of a sustained immune response in the brain is not exclusive from AD, and it’s seen as a central feature of neurodegenerative diseases. However, while general neuroinflammatory processes involve glial activation and cytokine dysregulation, AD-specific inflammation is characterized by microglial overactivation in response to amyloid B plaques, leading to a chronic inflammatory state (Kinney et al., 2018). It’s also seen that specific factors, such as gene modifications and insulin signalling impairment, plays a vital role in brain health and in AD’s course by promoting the deposition of Aβ plaques in specific regions of the brain. (Plum et al., 2005) The insulin receptors are found abundantly in the hippocampus and the cerebral cortex, which are the core areas related to AD pathology. The failure in it’s binding impairs neuronal development and plasticity, as well as learning and memory processes, being also associated with neuronal death. As this, patients with AD usually develop memory and learning impairment earlier then patients with other neurodegenerative diseases (Freude et al., 2009).

Nowadays, there is also the possibility of the early diagnosis of the disease due to advances in molecular tests and the use of neuroimaging markers, allowing it to be recognised even in the asymptomatic phase, which lasts between 10 and 20 years (Dubois et al., 2016, Scheltens et al., 2021). Specific biomarkers, such as p-Tau isoforms and amyloid proteins can be detected in different disease stages in plasma samples or in PET-ct. Also, AD is characterized by a reduction in Aβ_42_, the most amyloidogenic form of this peptide, and an increase in p-tau in the cerebrospinal fluid. The ratio of p-tau/Aβ_42_ levels is frequently used in AD diagnosis, having a high sensitivity and specificity when used along with the clinical features of the disease, being a tool to differentiate AD from other dementias (Studart Neto et al., 2024). In most cases, however, this disease is identified at stages where some cognitive decline is already present. Most of the diagnosis are made associating clinical tests and imaging exams. Neurocognitive tests can assess memory, attention and language impairment. In addition, imaging tests such as positron emission tomography (PET) and magnetic resonance imaging (MRI) can reveal typical brain changes of the disease (Schilling et al., 2022).

### Risk factors and modifiable contributors

Regarding risk factors for AD, although there are forms of presentation whose genesis is mediated by forms of autosomal dominant inheritance, the most prevalent form of AD is sporadic, which is largely related to environmental factors, being the main risk factor aging itself. Current research shows that more than 30 % of Alzheimer's cases can be linked to the presence of potentially modifiable risk factors. As this, the influence of educational level, lifestyle, social isolation and various comorbidities is equally important on the development of dementia, being comorbidities like hipertension, diabetes, obesity and dyslipidemia largely associated with it’s pathogenesis (Schilling et al., 2022). At same time, there is a high prevalence of dysbiosis and gastrointestinal disease in patients with AD, which has a strong association with increased beta-amyloid plaque formation and a chronic inflammatory state, being a potential risk factor which can contribute to neurodegeneration and be associated with the worsening of symptoms. Therefore, it is shown the importance of acting on these issues to prevent and delay the progression of cognitive decline associated with the pathology (Livingston et al., 2020).

### Current treatments and emerging strategies

The pharmacological treatments currently available for Alzheimer's disease primarily focus on mitigating symptoms, aiming to enhance patients' cognition, behavior, and overall quality of life. As this, acetylcholinesterase inhibitors are used to stimulate other preserved neurological pathways, once the deterioration of cholinergic neurons and the loss of neurotransmission are the major causes of the decline in cognitive function among these patients(Sharma, 2019). In moderate dementia, drugs such as N-methyl-aspartate (NMDA) receptor antagonists act on the pathological increase in glutamatergic pathways, which is also related to an improvement in the disease’s symptoms (Breijyeh and Karaman, 2020).

As a new approach, recent studies have been carried out to identify therapeutic targets aiming to both improve clinical outcomes and modify the disease trajectory. Among the drugs under investigation, monoclonal antibodies targeting amyloid beta protein deposition and inhibitors of Tau protein phosphorylation have emerged as prominent therapeutic agents. On other hand, even as these treatments have a relevant effect on senile plaque formation, they have not demonstrated proportional improvement in cognitive symptoms and significant changes in the clinical course of the disease (Avgerinos et al., 2021). This indicates the possible involvement of other factors such asmicroglia associated neuroinflammation, oxidative stress and dysfunction of the blood-brain barrier as an important part of AD’s pathophysiology. Therefore, the use of such drugs is still limited due to the large number of side effects compared to the small clinical benefit.

### Dietary interventions and probiotics

Dietary interventions have proven to be a promising complementary approach as they can be safely implemented and can act on the inflammatory state and important risk factors. Diets low in sodium, in simple carbohydrates and in ultra-processed foods, such as the DASH diet and the Mediterranean diet, have been shown to be effective in reducing the incidence of the disease by promoting better control of hypertension, dyslipidemia and diabetes. New dietary approaches such as the Mediterranean Intervention for Neurodegenerative Delay (MIND) diet, which in addition to controlling macronutrient intake, recommends the consumption of certain fruits, vegetables and legumes rich in substances with neuroprotective potential, such as green leafy vegetables and berries, have been shown to be even more effective in reducing the incidence of the dementia (Morris et al., 2015, Śliwińska and Jeziorek, 2021, van Soest et al., 2024).

In addition, the high prevalence of gut dysbiosis in patients with AD supports the hypothesis that an imbalance in the gut microbiota may be associated with disease progression through the activation of inflammatory pathways and increased oxidative stress, being frequently associated with the progression from Mild Cognitive Impairment (MCI) to AD. This imbalance can enhance glial cell reactivity, contributing to neuroinflammation, the activation of apoptotic mechanisms, and damage to the blood-brain barrier, ultimately resulting in neuronal degeneration and exacerbated synaptic dysfunction (Fig. 2). Supplementation with probiotics therefore has the potential to prevent the disease and delay dementia, as it can modulate the gut-brain axis by reducing NLRP3, TLR4 and MYD88 inflammatory pathways triggered by the overgrowth of pathogenic bacteria. As this, it may be possible to reduce oxidative stress and the release of IL-6, IL-B, IL8 and TNF-a, thereby reducing neuroinflammation. These interventions are generally well tolerated and have few contraindications, making them a viable option for many patients, complementing pharmacologic treatment and potentially improving quality of life (Liu et al., 2020).

### Neuroinflammation and microglial activation

The neuroinflammation is characterized by the release of pro-inflammatory cytokines such as IL-1β, IL-6, IL-8, and TNF, along with chemokines, complement system proteins, and small signaling molecules like prostaglandins, NO, and ROS. Acute inflammation in the brain has a protective function, acting as a response to injury, infection, or other harmful stimuli. However, the persistence of the inflammation leads to a chronic injury state, exacerbating the inflammatory response and causing neuronal damage. Microglia, immune cells residing in the central nervous system, play a central role in this process, being the primary cells responsible for brain inflammation. During pathological conditions, such as Alzheimer's disease (AD), microglia can shift from an anti-inflammatory (M2) state to a pro-inflammatory (M1) state, releasing cytokines and promoting synaptic loss, Tau phosphorylation, and memory loss. Alterations in microglial function are increasingly recognized as one of the central factors in the progression of neurodegenerative diseases like AD (Wang et al., 2023).

Interleukins 10 and 12 exhibit a reciprocal regulatory relationship in immune responses. IL-12, a potent pro-inflammatory cytokine, stimulates the production of interferon-gamma (IFN-γ) and promotes a Th1-type immune response. Conversely, IL-10 is a potent anti-inflammatory cytokine that inhibits the production of pro-inflammatory cytokines and suppresses immune cell activation. In the context of AD, the modulation of this IL-10/IL-12 axis represents a promising therapeutic strategy, particularly in mitigating neuroinflammation and microglial activation (Anwar et al., 2019, Ton et al., 2020).

### Kefir and neuroprotective potential

The composition of Kefir is a matrix of exopolysaccharides in which symbiotic lactic acid bacteria, acetic acid bacteria and yeast are found (Garofalo et al., 2020). The most common species of microorganisms present in kefir grains are *Candida kefyr*, *Kluyveromyces marxianus*s sp. Marxianus, *Lactobacillus acidophilus*, *Lactobacillus delbrueckii* subsp. Bulgarian, *Lactobacillus kefiranofaciens*, *Lacticaseibacillus paracasei*, *Lactiplantibacillus plantarum*, *Saccharomyces cerevisiae* and *Saccharomyces unisporus* (Prado et al., 2015).

During fermentation, lactic acid bacteria and yeast perform biochemical processes such as lactic acid fermentation, where lactose is converted into lactic acid and other organic acids. This lowers the milk's pH, coagulates proteins, and imparts a characteristic sour taste. Additionally, glycolysis converts glucose into pyruvate, which lactic acid bacteria metabolize into carbon dioxide (Egea et al., 2022, Sankhla et al., 2022). The metabolic activity of these microorganisms leads to the synthesis of proteins, peptides, essential amino acids, and B-complex vitamins necessary for cellular functions and the production of kefiran, a key polysaccharide (Arslan, 2015).

Kefiran, a water-soluble exopolysaccharide composed of galactose, glucose, and acetic acid, constitutes over half of the dry mass of kefir grains (Arslan, 2015, Moradi and Kalanpour, 2019). This compound functions as a prebiotic, supporting gut microbiota growth, and exhibits immunomodulatory, anti-inflammatory, antimicrobial, and antioxidant properties. These attributes contribute to the prevention of neurodegenerative disorders, as well as antitumor and curative effects(Moradi and Kalanpour, 2019).

The proteomic and peptidomic analysis of kefir revealed the presence of peptides with therapeutic potential for AD. These isolated peptides with up to ten amino acid residues exhibit antioxidant activity and inhibition of the enzyme acetylcholinesterase, a primary mechanism of action for globally recognized Alzheimer's treatments (Malta et al., 2022).

**Fig. 3:**
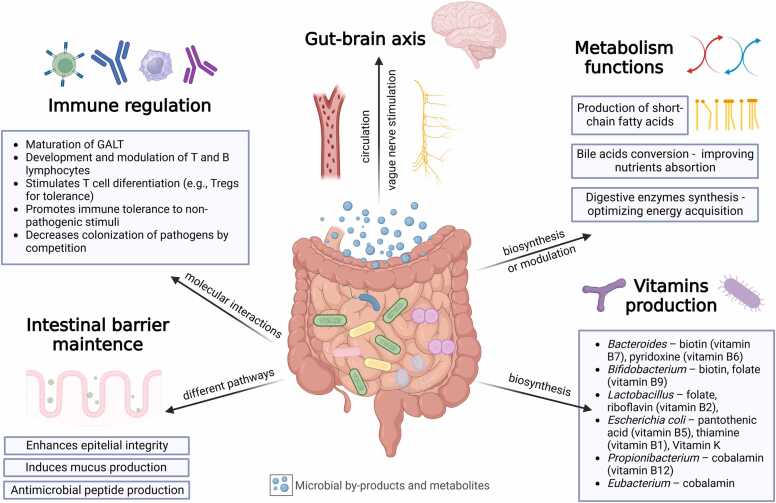
Fig. 2: GALT= Gut-associated lymphoid tissue. The figure illustrates the central role of the gut microbiota in human health through interconnected functions. It regulates the immune system by promoting tolerance to non-pathogenic stimuli and competing with pathogens. The microbiota affects the gut-brain axis through bidirectional nerve communication and bloodstream transport of bacterial metabolites, toxins, and fragments, influencing brain function and inflammation. It aids in metabolism by producing short-chain fatty acids, converting bile acids, and synthesizing digestive enzymes, while contributing to vitamin production. A healthy microbiota maintains the intestinal barrier, preventing harmful bacterial byproducts translocation to the bloodstream and protecting systemic and neurological health (LeBlanc et al., 2013). Created in https://BioRender.com.

The gastrointestinal microbiome plays an essential role in general homeostasis. Alterations in this microbiomecan lead to the development of multiple disorders, being often associated with neuropsychiatric diseases (Gulliver et al., 2022). Studies suggest that the intestinal microbiota influences inflammation, insulin, glucose and hepatic lipid metabolism, and also directly influences the central nervous system (CNS), as shown in Fig. 3, by modulating the endocrine system and gut-brain axis signalling pathways (Cox et al., 2014, Torres-Fuentes et al., 2017). Probiotics can also regulate innate and adaptive immune functions through interactions with epithelial or dendritic cells (Gulliver et al., 2022). These interactions promote anti-inflammatory immune responses of macrophages and T and B lymphocytes (Gulliver et al., 2022).

In this context, several studies point to the relationship between diet and gut microbiota in the aetiology of non-intestinal diseases such as neurodegenerative diseases, including AD (Anand et al., 2015, Gates et al., 2022, Jalilpiran et al., 2022, Ley et al., 2014). The composition of the gut microbiota and the decrease in microbial diversity have been linked to the aetiology of AD (Gates et al., 2022, Kesika et al., 2021, Yemula et al., 2021), demonstrating that gut-brain signalling plays an active role in the pathogenesis of neurodegenerative diseases (Gates et al., 2022).

Among the microorganisms present in Kefir, *Lactobacillus* species play a dual role in gut health. While certain strains of *Lactobacillus* can be abundant in dysbiosis and contribute to inflammation, the strains found in kefir have been shown to exert anti-inflammatory effects by producing metabolites that suppress harmful bacteria such as *Escherichia*. Furthermore, kefir promotes the growth of *Bacteroides*, a genus of anti-inflammatory bacteria, by creating a favorable gut environment through the production of short-chain fatty acids (SCFAs) like acetate, propionate, and butyrate (Stiemsma et al., 2020).

The cognitive benefits of the use of probiotic milk containing multiple *Lactobacillus* species was highlighted in multiple studies with AD patients (Zhang et al., 2023), being noticed by Akbari et al. (2016) and Zhang et al.(2023) an improvement in the cognitive function, measured by mini-mental state examination score, after a 12-week consumption (Akbari et al., 2016, Zhang et al., 2023). Also, the use of probiotics has shown potential to synergistically optimize the drug response, helping to maintain and/or recover the effectiveness of medications in patients with neurodegenerative diseases considered “refractory” (Pereira et al., 2021).

Continuous and long-term consumption of probiotics, including Kefir, has modulatory effects on proinflammatory biomarkers and antioxidant effects, and also contributes to the maintenance of neuronal networks (Akbari et al., 2016, Lemos et al., 2022, Radhouani et al., 2018, Ton et al., 2020). In this context, kefir supplementation supports the neuromodulatory process by mediating the neuroactive and neuroendocrine synthesis of acetylcholine, dopamine, serotonin, norepinephrine, adrenaline, glutamate, gamma-aminobutyric acid (GABA), and brain-derived neurotrophic factor (BDNF), as well as promoting the expression of their respective receptors (Akbari et al., 2016, Baj et al., 2019, Kowalski and Mulak, 2019, Martins et al., 2018).

On other hand, a randomized, double-blinded, and placebo-controlled clinical trial (n = 60) conducted by Agahi *et. al* (2018) didn’t find significant improvement on cognitive and biochemical markers in patients with severe AD with probiotic supplementation, which suggests that there are other factors that must be considered before starting a supplementary approach(Agahi et al., 2018).Also, the composition and homogeneity of Kefir microorganisms vary significantly depending on the region of production, which presents a significant limitation when comparing the results of different studies on its benefits.

## Conclusion

This systematic review shows that there are potential benefits for the use of kefir in AD management, as multiple advantages regarding inflammatory profile and symptom improvement were shown in invertebrates, rodents and humans studies. The gut-brain system shows an important role in AD pathogenesis, being evident that maintaining a healthy intestinal flora contributes to overall health, particularly for the nervous system. However, given the complexity and multifactorial nature of AD, more human studies should be conducted prioritizing comprehensive approaches that integrate pharmacological treatments, dietary modifications, and lifestyle interventions. Longitudinal studies and large-scale randomized clinical trials are needed to evaluate the long-term effects of probiotics like kefir, combined with other preventive measures, on cognitive decline. In this way, a more comprehensive understanding of the safety and efficacy of Kefir as an adjuvant can be achieved, providing clearer insights into its potential benefits and applications in clinical and therapeutic contexts.

## CRediT authorship contribution statement

**Mafra Ana Clara Morais:** Writing – original draft, Methodology, Investigation, Formal analysis. **Tanure Yuri Castelo Branco:** Writing – original draft, Investigation. **Brito Julio Cesar Moreira:** Writing – review & editing, Writing – original draft, Supervision, Project administration, Methodology, Investigation, Formal analysis, Data curation, Conceptualization. **Nascimento Israel Júnior Borges do:** Visualization, Conceptualization. **Fagundez Catherine:** Writing – review & editing. **Magalhães Rafael Coelho:** Writing – review & editing, Writing – original draft, Methodology, Investigation, Conceptualization. **Guimarães Bruna Luiza Motta:** Writing – original draft, Methodology, Investigation, Formal analysis.

## Declaration of Competing Interest

The authors declare that they have no known competing financial interests or personal relationships that could have appeared to influence the work reported in this paper.
