# Supplementary material for: Potential benefits of kefir and its compounds on Alzheimer's disease: A systematic review
Source: Brain Behav Immun Integr. 2025 Apr;10:100115. doi: 10.1016/j.bbii.2025.100115 (PMC12075057; doi:10.1016/j.bbii.2025.100115)
Supplement: Supplementary file 1 — Supplementary material [file mmc1.docx]

**Supplementary files**

**Potential Benefits of Kefir and its Compounds on Alzheimer**'**s Disease: A Systematic Review**

Yuri Castelo Branco Tanure^1^, Ana Clara Morais Mafra^1^, Bruna Luiza Motta Guimarães^1^, Rafael Coelho Magalhães^2^; Catherine Fagundez^3^; Israel Júnior Borges do Nascimento^4^, Julio Cesar Moreira Brito^1^

^1^Fundação Ezequiel Dias (FUNED), Belo Horizonte, MG, Brazil

^2^Universidade Federal de Minas Gerais (UFMG), Belo Horizonte, MG, Brazil

^3^Universidad de la República, Montevideo, Uruguay

^4^Division of Country Health Policies and Systems (CPS), World Health Organization, Regional Office for Europe, Copenhagen, 2100, Denmark

**Pubmed/MEDLINE**

((Neurodegenerative Diseases[Title/Abstract]) OR (Chronic Traumatic Encephalopathy[Title/Abstract]) OR (Heredodegenerative Disorders, Nervous System[Title/Abstract]) OR (Alexander Disease[Title/Abstract]) OR (Amyloid Neuropathies, Familial[Title/Abstract]) OR (Bulbo-Spinal Atrophy, X-Linked[Title/Abstract]) OR (Canavan Disease[Title/Abstract]) OR (Cockayne Syndrome[Title/Abstract]) OR (Dystonia Musculorum Deformans[Title/Abstract]) OR (Gerstmann-Straussler-Scheinker Disease[Title/Abstract]) OR (Hepatolenticular Degeneration[Title/Abstract]) OR (Hereditary Central Nervous System Demyelinating Diseases[Title/Abstract]) OR (Hereditary Sensory[Title/Abstract] AND Autonomic Neuropathies[Title/Abstract]) OR (Hereditary Sensory[Title/Abstract] AND Motor Neuropathy[Title/Abstract]) OR (Huntington Disease[Title/Abstract]) OR (Lafora Disease[Title/Abstract]) OR (Myotonia Congenita[Title/Abstract]) OR (Myotonic Dystrophy[Title/Abstract]) OR (Neurofibromatoses[Title/Abstract]) OR (Neuronal Ceroid-Lipofuscinoses[Title/Abstract]) OR (Optic Atrophies, Hereditary[Title/Abstract]) OR (Pantothenate Kinase-Associated Neurodegeneration[Title/Abstract]) OR (Spinal Muscular Atrophies of Childhood[Title/Abstract]) OR (Spinocerebellar Degenerations[Title/Abstract]) OR (Tourette Syndrome[Title/Abstract]) OR (Tuberous Sclerosis[Title/Abstract]) OR (Unverricht-Lundborg Syndrome[Title/Abstract]) OR (Motor Neuron Disease[Title/Abstract]) OR (Amyotrophic Lateral Sclerosis[Title/Abstract]) OR (Bulbar Palsy, Progressive[Title/Abstract]) OR (Muscular Atrophy, Spinal[Title/Abstract]) OR (Paraneoplastic Syndromes, Nervous System[Title/Abstract]) OR (Anti-N-Methyl-D-Aspartate Receptor Encephalitis[Title/Abstract]) OR (Limbic Encephalitis[Title/Abstract]) OR (Myasthenia Gravis[Title/Abstract]) OR (Myelitis, Transverse[Title/Abstract]) OR (Opsoclonus-Myoclonus Syndrome[Title/Abstract]) OR (Paraneoplastic Cerebellar Degeneration[Title/Abstract]) OR (Paraneoplastic Polyneuropathy[Title/Abstract]) OR (Postpoliomyelitis Syndrome[Title/Abstract]) OR (Prion Diseases[Title/Abstract]) OR (Encephalopathy, Bovine Spongiform[Title/Abstract]) OR (Gerstmann-Straussler-Scheinker Disease[Title/Abstract]) OR (Insomnia, Fatal Familial[Title/Abstract]) OR (Kuru[Title/Abstract]) OR (Scrapie[Title/Abstract]) OR (Wasting Disease, Chronic[Title/Abstract]) OR (Subacute Combined Degeneration[Title/Abstract]) OR (Synucleinopathies[Title/Abstract]) OR (Lewy Body Disease[Title/Abstract]) OR (Multiple System Atrophy[Title/Abstract]) OR (Parkinson Disease[Title/Abstract]) OR (Tauopathies[Title/Abstract]) OR (Alzheimer Disease[Title/Abstract]) OR (Corticobasal Degeneration[Title/Abstract]) OR (Diffuse Neurofibrillary Tangles with Calcification[Title/Abstract]) OR (Supranuclear Palsy, Progressive[Title/Abstract]) OR (TDP-43 Proteinopathies[Title/Abstract]) OR (Amyotrophic Lateral Sclerosis[Title/Abstract]) OR (dementia[Title/Abstract]) OR (alzheimer[Title/Abstract])) AND (kefir[Title/Abstract])

**Scopus**

TITLE-ABS-KEY ( ( ( Neurodegenerative Diseases ) OR ( Chronic Traumatic Encephalopathy ) OR ( Heredodegenerative Disorders, Nervous System ) OR ( Alexander Disease ) OR ( Amyloid Neuropathies, Familial ) OR ( Bulbo-Spinal Atrophy, X-Linked ) OR ( Canavan Disease ) OR ( Cockayne Syndrome ) OR ( Dystonia Musculorum Deformans ) OR ( Gerstmann-Straussler-Scheinker Disease ) OR ( Hepatolenticular Degeneration ) OR ( Hereditary Central Nervous System Demyelinating Diseases ) OR ( Hereditary Sensory AND Autonomic Neuropathies ) OR ( Hereditary Sensory AND Motor Neuropathy ) OR ( Huntington Disease ) OR ( Lafora Disease ) OR ( Myotonia Congenita ) OR ( Myotonic Dystrophy ) OR ( Neurofibromatoses ) OR ( Neuronal Ceroid-Lipofuscinoses ) OR ( Optic Atrophies, Hereditary ) OR ( Pantothenate Kinase-Associated Neurodegeneration ) OR ( Spinal Muscular Atrophies of Childhood ) OR ( Spinocerebellar Degenerations ) OR ( Tourette Syndrome ) OR ( Tuberous Sclerosis ) OR ( Unverricht-Lundborg Syndrome ) OR ( Motor Neuron Disease ) OR ( Amyotrophic Lateral Sclerosis ) OR ( Bulbar Palsy, Progressive ) OR ( Muscular Atrophy, Spinal ) OR ( Paraneoplastic Syndromes, Nervous System ) OR ( Anti-N-Methyl-D-Aspartate Receptor Encephalitis ) OR ( Limbic Encephalitis ) OR ( Myasthenia Gravis ) OR ( Myelitis, Transverse ) OR ( Opsoclonus-Myoclonus Syndrome ) OR ( Paraneoplastic Cerebellar Degeneration ) OR ( Paraneoplastic Polyneuropathy ) OR ( Postpoliomyelitis Syndrome ) OR ( Prion Diseases ) OR ( Encephalopathy, Bovine Spongiform ) OR ( Gerstmann-Straussler-Scheinker Disease ) OR ( Insomnia, Fatal Familial ) OR ( Kuru ) OR ( Scrapie ) OR ( Wasting Disease, Chronic ) OR ( Subacute Combined Degeneration ) OR ( hemoglobinopathies ) OR ( Lewy Body Disease ) OR ( Multiple System Atrophy ) OR ( Parkinson Disease ) OR ( Tauopathies ) OR ( Alzheimer Disease ) OR ( Corticobasal Degeneration ) OR ( Diffuse Neurofibrillary Tangles with Calcification ) OR ( Supranuclear Palsy, Progressive ) OR ( TDP-43 retinopathies ) OR ( Amyotrophic Lateral Sclerosis ) OR ( dementia ) OR ( alzheimer ) ) AND ( kefir ) )

**EMBASE**

('neurodegenerative diseases':ti,ab,kw OR 'chronic traumatic encephalopathy':ti,ab,kw OR 'heredodegenerative disorders, nervous system':ti,ab,kw OR 'alexander disease':ti,ab,kw OR 'amyloid neuropathies, familial':ti,ab,kw OR 'bulbo-spinal atrophy, x-linked':ti,ab,kw OR 'canavan disease':ti,ab,kw OR 'cockayne syndrome':ti,ab,kw OR 'dystonia musculorum deformans':ti,ab,kw OR 'hepatolenticular degeneration':ti,ab,kw OR 'hereditary central nervous system demyelinating diseases':ti,ab,kw OR ('hereditary sensory':ti,ab,kw AND 'autonomic neuropathies':ti,ab,kw) OR ('hereditary sensory':ti,ab,kw AND 'motor neuropathy':ti,ab,kw) OR 'huntington disease':ti,ab,kw OR 'lafora disease':ti,ab,kw OR 'myotonia congenita':ti,ab,kw OR 'myotonic dystrophy':ti,ab,kw OR neurofibromatoses:ti,ab,kw OR 'neuronal ceroid-lipofuscinoses':ti,ab,kw OR 'optic atrophies, hereditary':ti,ab,kw OR 'pantothenate kinase-associated neurodegeneration':ti,ab,kw OR 'spinal muscular atrophies of childhood':ti,ab,kw OR 'spinocerebellar degenerations':ti,ab,kw OR 'tourette syndrome':ti,ab,kw OR 'tuberous sclerosis':ti,ab,kw OR 'unverricht-lundborg syndrome':ti,ab,kw OR 'motor neuron disease':ti,ab,kw OR 'bulbar palsy, progressive':ti,ab,kw OR 'muscular atrophy, spinal':ti,ab,kw OR 'paraneoplastic syndromes, nervous system':ti,ab,kw OR 'anti-n-methyl-d-aspartate receptor encephalitis':ti,ab,kw OR 'limbic encephalitis':ti,ab,kw OR 'myasthenia gravis':ti,ab,kw OR 'myelitis, transverse':ti,ab,kw OR 'opsoclonus-myoclonus syndrome':ti,ab,kw OR 'paraneoplastic cerebellar degeneration':ti,ab,kw OR 'paraneoplastic polyneuropathy':ti,ab,kw OR 'postpoliomyelitis syndrome':ti,ab,kw OR 'prion diseases':ti,ab,kw OR 'encephalopathy, bovine spongiform':ti,ab,kw OR 'gerstmann-straussler-scheinker disease':ti,ab,kw OR 'insomnia, fatal familial':ti,ab,kw OR kuru:ti,ab,kw OR scrapie:ti,ab,kw OR 'wasting disease, chronic':ti,ab,kw OR 'subacute combined degeneration':ti,ab,kw OR hemoglobinopathies:ti,ab,kw OR 'lewy body disease':ti,ab,kw OR 'multiple system atrophy':ti,ab,kw OR 'parkinson disease':ti,ab,kw OR tauopathies:ti,ab,kw OR 'alzheimer disease':ti,ab,kw OR 'corticobasal degeneration':ti,ab,kw OR 'diffuse neurofibrillary tangles with calcification':ti,ab,kw OR 'supranuclear palsy, progressive':ti,ab,kw OR 'tdp-43 retinopathies':ti,ab,kw OR 'amyotrophic lateral sclerosis':ti,ab,kw OR dementia:ti,ab,kw OR alzheimer:ti,ab,kw) AND kefir:ti,ab,kw

**BVS**

( ( ( neurodegenerative diseases ) OR ( chronic traumatic encephalopathy ) OR ( heredodegenerative disorders, nervous system ) OR ( alexander disease ) OR ( amyloid neuropathies, familial ) OR ( bulbo-spinal atrophy, x-linked ) OR ( canavan disease ) OR ( cockayne syndrome ) OR ( dystonia musculorum deformans ) OR ( gerstmann-straussler-scheinker disease ) OR ( hepatolenticular degeneration ) OR ( hereditary central nervous system demyelinating diseases ) OR ( hereditary sensory AND autonomic neuropathies ) OR ( hereditary sensory AND motor neuropathy ) OR ( huntington disease ) OR ( lafora disease ) OR ( myotonia congenita ) OR ( myotonic dystrophy ) OR ( neurofibromatoses ) OR ( neuronal ceroid-lipofuscinoses ) OR ( optic atrophies, hereditary ) OR ( pantothenate kinase-associated neurodegeneration ) OR ( spinal muscular atrophies of childhood ) OR ( spinocerebellar degenerations ) OR ( tourette syndrome ) OR ( tuberous sclerosis ) OR ( unverricht-lundborg syndrome ) OR ( motor neuron disease ) OR ( amyotrophic lateral sclerosis ) OR ( bulbar palsy, progressive ) OR ( muscular atrophy, spinal ) OR ( paraneoplastic syndromes, nervous system ) OR ( anti-n-methyl-d-aspartate receptor encephalitis ) OR ( limbic encephalitis ) OR ( myasthenia gravis ) OR ( myelitis, transverse ) OR ( opsoclonus-myoclonus syndrome ) OR ( paraneoplastic cerebellar degeneration ) OR ( paraneoplastic polyneuropathy ) OR ( postpoliomyelitis syndrome ) OR ( prion diseases ) OR ( encephalopathy, bovine spongiform ) OR ( gerstmann-straussler-scheinker disease ) OR ( insomnia, fatal familial ) OR ( kuru ) OR ( scrapie ) OR ( wasting disease, chronic ) OR ( subacute combined degeneration ) OR ( hemoglobinopathies ) OR ( lewy body disease ) OR ( multiple system atrophy ) OR ( parkinson disease ) OR ( tauopathies ) OR ( alzheimer disease ) OR ( corticobasal degeneration ) OR ( diffuse neurofibrillary tangles with calcification ) OR ( supranuclear palsy, progressive ) OR ( tdp-43 retinopathies ) OR ( amyotrophic lateral sclerosis ) OR ( dementia ) OR ( alzheimer ) ) AND ( kefir ) )

**Web of Science**

( ( ( neurodegenerative diseases ) OR ( chronic traumatic encephalopathy ) OR ( heredodegeneration disorders, nervous system ) OR ( alexander disease ) OR ( amyloid neuropathies, familial ) OR ( bulbo-spinal atrophy, x-linked ) OR ( canavan disease ) OR ( cockayne syndrome ) OR ( dystonia musculorum deformans ) OR ( gerstmann-straussler-scheinker disease ) OR ( hepatolenticular degeneration ) OR ( hereditary central nervous system demyelinating diseases ) OR ( hereditary sensory AND autonomic neuropathies ) OR ( hereditary sensory AND motor neuropathy ) OR ( huntington disease ) OR ( lafora disease ) OR ( myotonia congenita ) OR ( myotonic dystrophy ) OR ( neurofibromatosis ) OR ( neuronal ceroid-lipofuscinoses ) OR ( optic atrophies, hereditary ) OR ( pantothenate kinase-associated neurodegeneration ) OR ( spinal muscular atrophies of childhood ) OR ( spinocerebellar degenerations ) OR ( tourette syndrome ) OR ( tuberous sclerosis ) OR ( unverricht-lundborg syndrome ) OR ( motor neuron disease ) OR ( amyotrophic lateral sclerosis ) OR ( bulbar palsy, progressive ) OR ( muscular atrophy, spinal ) OR ( paraneoplastic syndromes, nervous system ) OR ( anti-n-methyl-d-aspartate receptor encephalitis ) OR ( limbic encephalitis ) OR ( myasthenia gravis ) OR ( myelitis, transverse ) OR ( opsoclonus-myoclonus syndrome ) OR ( paraneoplastic cerebellar degeneration ) OR ( paraneoplastic polyneuropathy ) OR ( postpoliomyelitic syndrome ) OR ( prion diseases ) OR ( encephalopathy, bovine spongiform ) OR ( gerstmann-straussler-scheinker disease ) OR ( insomnia, fatal familial ) OR ( kuru ) OR ( scrapie ) OR ( wasting disease, chronic ) OR ( subacute combined degeneration ) OR ( hemoglobinopathies ) OR ( lewy body disease ) OR ( multiple system atrophy ) OR ( parkinson disease ) OR ( tauopathies ) OR ( alzheimer disease ) OR ( corticobasal degeneration ) OR ( diffuse neurofibrillary tangles with calcification ) OR ( supranuclear palsy, progressive ) OR ( tdp-43 retinopathies ) OR ( amyotrophic lateral sclerosis ) OR ( dementia ) OR ( alzheimer ) ) AND ( kefir ) ) (Abstract) or ( ( ( neurodegenerative diseases ) OR ( chronic traumatic encephalopathy ) OR ( heredodegeneration disorders, nervous system ) OR ( alexander disease ) OR ( amyloid neuropathies, familial ) OR ( bulbo-spinal atrophy, x-linked ) OR ( canavan disease ) OR ( cockayne syndrome ) OR ( dystonia musculorum deformans ) OR ( gerstmann-straussler-scheinker disease ) OR ( hepatolenticular degeneration ) OR ( hereditary central nervous system demyelinating diseases ) OR ( hereditary sensory AND autonomic neuropathies ) OR ( hereditary sensory AND motor neuropathy ) OR ( huntington disease ) OR ( lafora disease ) OR ( myotonia congenita ) OR ( myotonic dystrophy ) OR ( neurofibromatosis ) OR ( neuronal ceroid-lipofuscinoses ) OR ( optic atrophies, hereditary ) OR ( pantothenate kinase-associated neurodegeneration ) OR ( spinal muscular atrophies of childhood ) OR ( spinocerebellar degenerations ) OR ( tourette syndrome ) OR ( tuberous sclerosis ) OR ( unverricht-lundborg syndrome ) OR ( motor neuron disease ) OR ( amyotrophic lateral sclerosis ) OR ( bulbar palsy, progressive ) OR ( muscular atrophy, spinal ) OR ( paraneoplastic syndromes, nervous system ) OR ( anti-n-methyl-d-aspartate receptor encephalitis ) OR ( limbic encephalitis ) OR ( myasthenia gravis ) OR ( myelitis, transverse ) OR ( opsoclonus-myoclonus syndrome ) OR ( paraneoplastic cerebellar degeneration ) OR ( paraneoplastic polyneuropathy ) OR ( postpoliomyelitic syndrome ) OR ( prion diseases ) OR ( encephalopathy, bovine spongiform ) OR ( gerstmann-straussler-scheinker disease ) OR ( insomnia, fatal familial ) OR ( kuru ) OR ( scrapie ) OR ( wasting disease, chronic ) OR ( subacute combined degeneration ) OR ( hemoglobinopathies ) OR ( lewy body disease ) OR ( multiple system atrophy ) OR ( parkinson disease ) OR ( tauopathies ) OR ( alzheimer disease ) OR ( corticobasal degeneration ) OR ( diffuse neurofibrillary tangles with calcification ) OR ( supranuclear palsy, progressive ) OR ( tdp-43 retinopathies ) OR ( amyotrophic lateral sclerosis ) OR ( dementia ) OR ( alzheimer ) ) AND ( kefir ) ) (Title)
